# Unveiling the Unknown: Nicaragua’s First Recorded Case of Mayer-Rokitansky-Küster-Hauser Syndrome

**DOI:** 10.7759/cureus.72895

**Published:** 2024-11-02

**Authors:** María Esther Suárez Garcia, Andres Rivera, Carlo Marcelo Vargas Salgado, Christopher Romero, Lorenzo E Aragón Conrado, Catherine S Moreno Cabrera

**Affiliations:** 1 Obstetrics and Gynecology, Hospital Militar Escuela "Dr. Alejandro Dávila Bolaños", Managua, NIC; 2 School of Medicine, Hospital Militar Escuela "Dr. Alejandro Dávila Bolaños", Managua, NIC

**Keywords:** co2 laser vaginoplasty, mayer-rokitansky-küster-hauser syndrome, multidisciplinary management, primary amenorrhea, uterovaginal hypoplasia

## Abstract

Mayer-Rokitansky-Küster-Hauser (MRKH) syndrome is a congenital disorder affecting the female reproductive system, primarily characterized by the absence or underdevelopment of the uterus and upper two-thirds of the vagina, with preserved ovarian function and normal secondary sexual characteristics. It is a rare disease though prevalence may vary based on genetic and environmental factors. This report details a case of a 26-year-old female patient with a history of smoking, alcohol use, and prior inguinal hernioplasty, presenting with primary amenorrhea and inability to engage in vaginal intercourse. The physical examination revealed signs of androgenic acne, acanthosis in the breasts, Tanner stage 4 breast development, and a reduced clitoral hood. An imperforate hymen was confirmed upon clinical examination. Pelvic ultrasound showed a hypoplastic uterus. Laboratory findings indicated hypogonadotropic hypogonadism, and genetic testing ruled out Turner syndrome and imperforate hymen. The next step was magnetic resonance imaging (MRI) which confirmed uterine hypoplasia and vaginal too, leading to the diagnosis of MRKH syndrome. The patient underwent successful CO2 laser vaginoplasty, and psychological support was provided to address the emotional and social aspects related to the diagnosis. This case, being the first documented in Nicaragua, highlights the importance of early diagnosis and a personalized treatment approach that addresses both the physical and emotional aspects of patients. Additionally, it underscores the need for close collaboration between various specialties including gynecology, endocrinology, genetics, and psychology to ensure comprehensive and optimal clinical and emotional outcomes in the management of patients with MRKH syndrome.

## Introduction

Mayer-Rokitansky-Küster-Hauser (MRKH) syndrome is a congenital anomaly of the female reproductive tract, primarily characterized by the absence or underdevelopment of the uterus and the upper two-thirds of the vagina, while the ovaries and secondary sexual characteristics remain normal. The prevalence of this syndrome is approximately 1 in 4,500 live female births, although this figure may vary among different populations due to genetic and environmental factors [1].

Although most cases of MRKH are sporadic in genetics, families with multiple affected individuals have been reported, suggesting a hereditary component in certain subgroups. Mutations in genes such as WNT4, LHX1, and HNF1B have been associated with the syndrome’s development, although the etiology is complex and likely involves interactions with epigenetic factors [2]. Ongoing research in this field remains crucial for developing more personalized treatments in the future.

## Case presentation

A 26-year-old female patient with a history of smoking and alcohol use, as well as left inguinal hernioplasty at age 6, and being overweight with a body mass index of 27.3, presents with primary amenorrhea, having never experienced menstruation in her lifetime. She reports engaging in non-penetrative sexual activity and has used hormonal methods, including cyproterone and ethinylestradiol, for two months without response.

Physical examination reveals hypopigmentation in the left submental area and androgenic acne. Breast examination shows Tanner's stage 4 development, with fibrocystic consistency and no galactorrhea. Acanthosis is present on both breasts. Genital examination reveals a reduced clitoral hood size and a small external urethral meatus. Upon performing the Valsalva maneuver, an imperforate hymen is noted, confirmed by swab insertion and palpation. 

**Figure 1:**
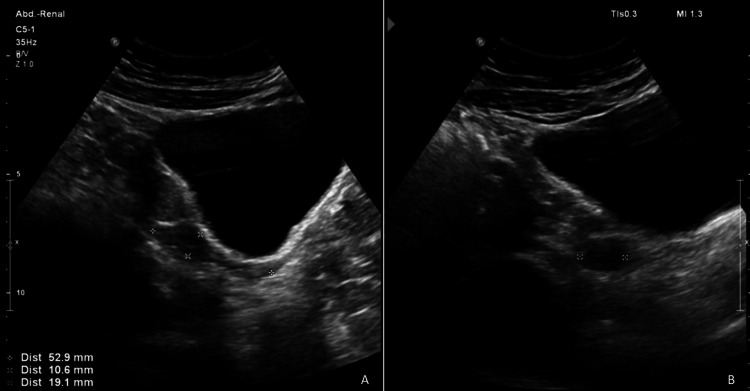
Pelvic Ultrasound (A and B) The uterus is anteflexed, with regular, well-defined contours. The dimensions are 53 mm in the longitudinal axis, 10 mm in the anteroposterior axis, and 19 mm in the transverse axis. The endometrium is central, echogenic, and measures 3 mm.

An ultrasound was performed, revealing that the uterus is reduced in size for the patient’s age (Figures 1A, 1B).

The endocrinology department conducted laboratory tests, which revealed hypogonadotropic hypogonadism (Table 1). Due to thyroid dysfunction, levothyroxine was initiated at 12.5 micrograms. The patient was referred to genetics to explore differential diagnoses, including Turner syndrome mosaicism and imperforate hymen. Karyotype testing was recommended and revealed a normal 46 XX karyotype, ruling out these conditions.

**Table 1:** Laboratory Tests Performed with Results and Reference Values FSH: Follicle-Stimulating Hormone; LH: Luteinizing Hormone; TSH: Thyroid-Stimulating Hormone; FT4: Free Thyroxine; FT3: Free Triiodothyronine; CA-125: Carbohydrate Antigen 125; CA 19-9: Carbohydrate Antigen 19-9

Laboratory Tests	Results	Reference Range
Hematocrit	36.1%	36%-43%
Hemoglobin	12.6 g/dL	12-16 g/dL
White Blood Cell Count	8.77 x 10³/µL	5-10 x 10³/µL
Neutrophils	53%	55-65%
Lymphocytes	38.4%	25-35%
Platelets	336 x 10³/µL	150-500 10³/µL
Carbohydrate Antigen 125 (CA-125)	6.98 U/mL	<35 U/mL
Carbohydrate Antigen 19-9 (CA 19-9)	2.53 U/mL	<37 U/mL
Estradiol	4.2 ng/mL	30-400 pg/mL
Follicle-Stimulating Hormone (FSH)	5.11 mIU/mL	3-10 mUI/ml
Luteinizing Hormone (LH)	2.9 mIU/mL	1.9-12.5 mIU/mL
Progesterone	<0.050 ng/mL	0.1-1.5 ng/mL
Prolactin	12.5 ng/mL	4.1-24.1 ng / ml
Free Thyroxine (FT4)	0.846 ng/dL	0.93-1.7 ng/dL
Free Triiodothyronine (FT3)	3.41 pg/mL	2.0-4.4 pg/mL
Thyroid-Stimulating Hormone (TSH)	2.77 µIU/mL	0.4-4.0 µIU/mL
Estradiol	<5.00 pg/mL	30-400 pg/mL
Glucose	87.12 mg/dL	74-106 mg/dL

Two months later, the patient was re-evaluated by the endocrinology department, with new laboratory results showing prolactin at 11 ng/mL and free thyroxine (FT4) at 0.86 ng/dL.

**Figure 2:**
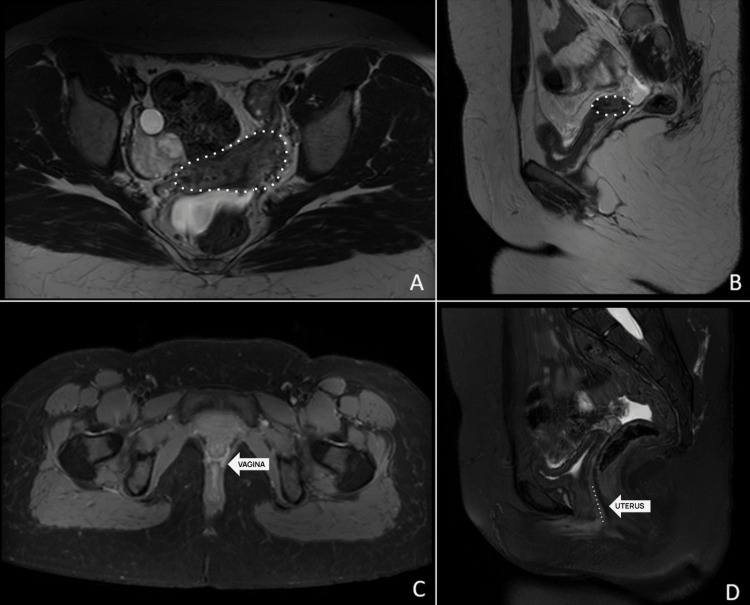
Pelvic Magnetic Resonance Imaging (A) Axial T2, (B) Sagittal T2, (C) Axial T1 with Weighted Image and Contrast, and (D) Sagittal T2 Weighted Image (A) A white-outlined area with a hypoplastic uterus is observed, with a small rudimentary uterine structure and no discernible endometrial cavity. (B) A white-circumscribed area confirms the presence of a small, underdeveloped uterine remnant, lacking an endometrial stripe and distinct uterine layers. The uterine body appears hypointense, without normal anatomical landmarks. (C) The arrow points to the vagina, identified as the H-shaped hyperintense structure. It appears small, with poor contrast enhancement, indicating underdevelopment (D) The arrow points to a white straight line that reflects the path of the hypoplastic uterus located posterior to the urethra. This line clearly delineates the hypoplastic uterine remnant from the surrounding pelvic fat, with no evidence of functional endometrial tissue in the underdeveloped uterine structure.

A pituitary MRI was ordered to investigate secondary amenorrhea, but no structural abnormalities were found. However, a pelvic MRI revealed uterine hypoplasia (Figures 2A-2D), confirming the diagnosis of MRKH syndrome. 

**Figure 3:**
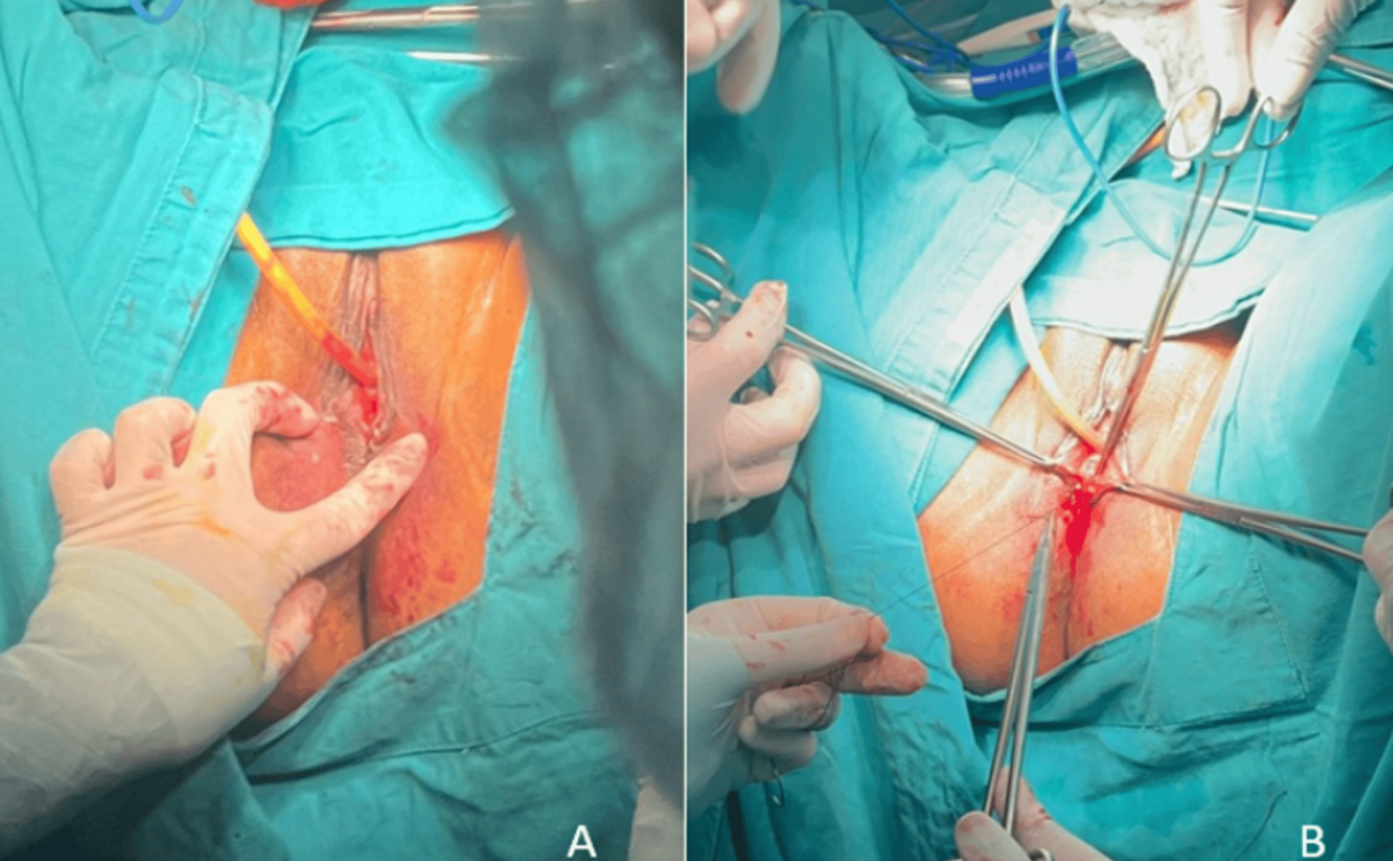
(A, B) CO2 Laser Vaginoplasty

In collaboration with the urogynecology service, a CO2 laser vaginoplasty was performed (Figures 3A, 3B), which was completed without complications. The patient was able to ambulate within 24 hours post-surgery.

**Figure 4:**
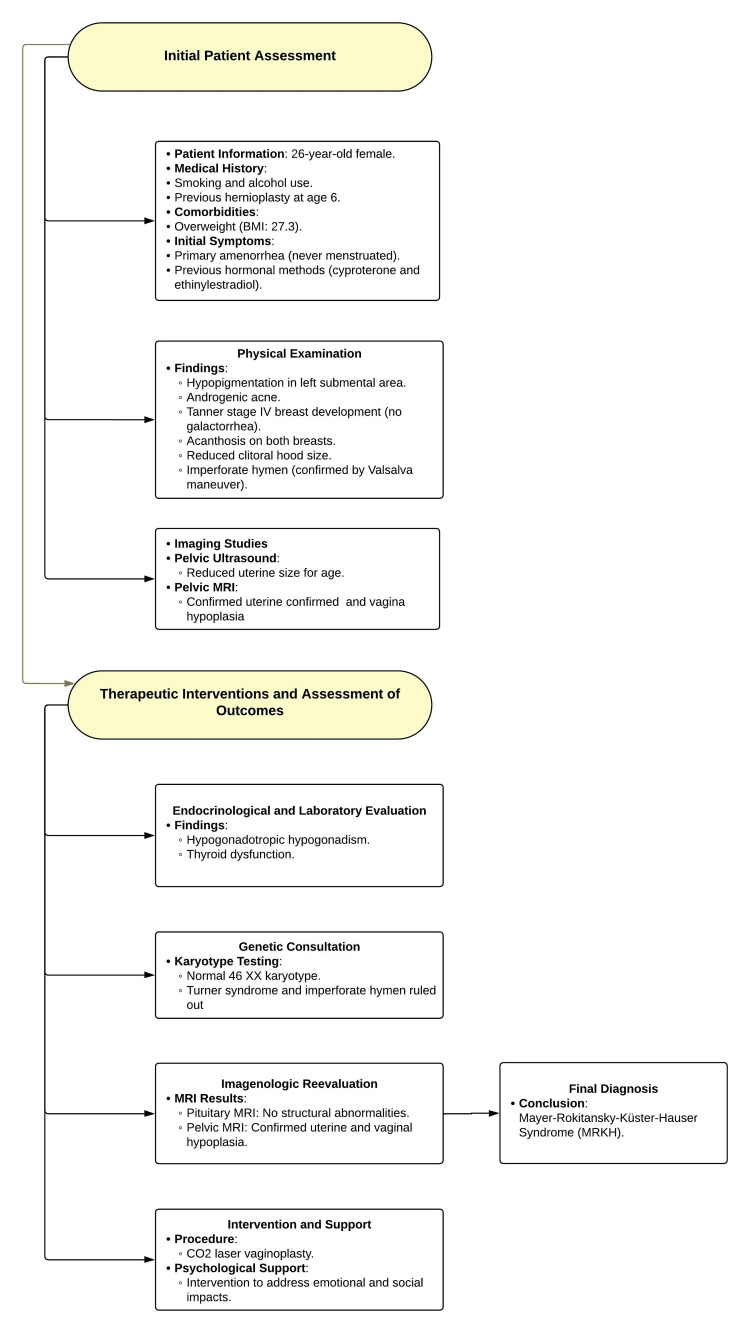
Flowchart of Diagnosis, Management, and Follow-up

Psychological support was provided due to the potential impact of this condition on personal identity, perceptions of fertility, and sexual life. The psychological intervention helped the patient cope with the emotional and social challenges associated with the diagnosis, significantly improving her emotional well-being (Figure 4).

## Discussion

The female reproductive tract in humans consists of the fallopian tubes, uterus, cervix, and vagina. While the lower portion of the vagina develops from the urogenital sinus, the remaining structures, such as the uterus and fallopian tubes, originate from the paramesonephric (Müllerian) ducts [3]. Disruptions in the normal embryologic development of these reproductive structures, particularly the Müllerian ducts, can result in a range of congenital anomalies of the female reproductive tract [4]. Development of the female reproductive system begins around the third week of embryogenesis. Importantly, the development of the gonads occurs independently from the genital tract, which explains why individuals with uterovaginal anomalies may still have normal ovaries with appropriate hormonal function [5].

Congenital anomalies of the reproductive organs have been classified multiple times by various gynecological bodies; however, the gold standard in the United States is the classification system provided by the American Society for Reproductive Medicine (ASRM), which incorporates both Müllerian defects and previously excluded anatomical variations [6].

MRKH syndrome, also known as Müllerian agenesis or congenital absence of the uterus and vagina, is a disorder characterized by the absence or incomplete development of the uterus and upper portion of the vagina in women with a normal female karyotype (46, XX). Despite the typical morphology of external genitalia, patients with MRKH syndrome retain normal gonadal endocrine function, allowing for puberty to occur with the development of breast and pubic hair. This syndrome most commonly presents during adolescence as primary amenorrhea and accounts for approximately 16% of such cases, second only to ovarian dysfunction [7].

Müllerian agenesis is frequently associated with extragenital malformations, primarily affecting the renal and skeletal systems. Epidemiologically, the prevalence of vaginal agenesis is cited as 1 in 5000 females, with estimates ranging from 1 in 4000 to 1 in 10,000 women [8]. Mayer was first described in 1829 and later expanded upon by Rokitansky, Küster, Hauser, and Schreiner, leading to its current designation as MRKH syndrome [9]. The syndrome is classified into two main subtypes: type I, characterized by isolated uterovaginal agenesis, and type II, which involves more extensive extragenital anomalies such as renal agenesis, horseshoe kidney, and skeletal malformations [10].

Genetic studies have identified a low prevalence of point mutations in genes such as WNT4, HNF1B, and LHX1 in individuals with MRKH, although most cases are sporadic. Epigenetic and environmental factors, including fetal exposure to diethylstilbestrol (DES) and other compounds like organotin and phthalates, have also been implicated in the syndrome's development. Despite these findings, familial occurrences suggest that genetic factors likely play a role in the pathogenesis of MRKH [11,12].

Diagnosis involves clinical evaluation, including a thorough physical examination, imaging studies such as ultrasound and magnetic resonance imaging (MRI), as well as genetic karyotyping to exclude individuals with a 46, XY profile. Patients with MRKH typically have a normal female karyotype (46, XX) and functional ovaries, allowing them to develop secondary sexual characteristics. Physical examination should include an assessment of the external genitalia and vaginal examination (which is contraindicated in prepubertal adolescents), usually revealing normal external genitalia with a vaginal "dimple" or a small cavity. Transperineal or transabdominal ultrasound often demonstrates the absence of the uterus and the presence of ovaries [13].

MRI of the internal genitalia is considered the gold standard for diagnosing uterovaginal agenesis in MRKH syndrome and should be performed whenever available. MRI offers superior detail in visualizing Müllerian structures (whether remnants or complete agenesis) and can identify the presence of endometrium in uterine remnants. MRI also visualizes the ovaries and associated extragenital malformations, and its high interrater reliability often renders laparoscopy unnecessary except in cases where symptomatic uterine remnants require surgical removal [14]. Complementary studies, such as ultrasound or MRI, are used to detect related malformations, including renal and skeletal anomalies, while screening for auditory and cardiac abnormalities is considered based on clinical findings [15].

Chromosomal analysis is frequently performed to confirm a normal female karyotype (46, XX). Several differential diagnoses should be excluded, as Müllerian agenesis is sometimes erroneously reported in 46, XX and 45, X females with ovarian insufficiency (gonadal dysgenesis) and estrogen deficiency. However, exogenous estrogen exposure has been reported to induce uterine development in these patients, suggesting absent uterine development at puberty rather than true agenesis [16].

The first-line treatment for vaginal agenesis is non-surgical, utilizing vaginal dilators [17]. If non-surgical therapy fails or the patient opts for a surgical approach, options include the McIndoe procedure, sigmoid vaginoplasty, and the Davydov procedure, among others [3].

## Conclusions

This case exemplifies a variant of MRKH syndrome, typically characterized by uterovaginal agenesis; however, in this instance, there was hypoplasia of both tissues, with preservation of ovarian function. The patient presented with primary amenorrhea, a clinical presentation that encompasses multiple differential diagnoses. This prompted a multidisciplinary evaluation that included the endocrinology service for hormonal studies, the genetics service for karyotype analysis, and imaging studies via magnetic resonance, which confirmed the diagnosis of uterine hypoplasia and vaginal hypoplasia.

The surgical intervention was successfully performed by the urogynecology service using CO2 laser-assisted vaginoplasty, achieving adequate anatomical reconstruction with no immediate postoperative complications. Additionally, therapeutic management was complemented with psychological support, essential for addressing the emotional and social impact that this diagnosis can generate in the patient, highlighting the importance of a multidisciplinary approach in managing the condition. In a developing country like Nicaragua, this case underscores the significance of early diagnosis and personalized treatment. It also emphasizes the need for close collaboration among multiple specialties to ensure comprehensive management that addresses both physical and emotional aspects, to improve the quality of life for those living with this condition.

## References

[1] Morcel K, Camborieux L, Guerrier D (2007). Mayer-Rokitansky-Küster-Hauser (MRKH) syndrome. Orphanet J Rare Dis.

[2] Williams LS, Demir Eksi D, Shen Y (2017). Genetic analysis of Mayer-Rokitansky-Kuster-Hauser syndrome in a large cohort of families. Fertil Steril.

[3] Laufer MR (2024). Congenital Anomalies of the Hymen and Vagina. UpToDate.

[4] Voutilainen R (1992). Differentiation of the fetal gonad. Horm Res.

[5] Garrett LA, Vargas SO, Drapkin R, Laufer MR (2008). Does the fimbria have an embryologic origin distinct from that of the rest of the fallopian tube?. Fertil Steril.

[6] Pfeifer SM, Attaran M, Goldstein J (2021). ASRM müllerian anomalies classification 2021. Fertil Steril.

[7] Griffin JE, Edwards C, Madden JD, Harrod MJ, Wilson JD (1976). Congenital absence of the vagina. The Mayer-Rokitansky-Kuster-Hauser syndrome. Ann Intern Med.

[8] Herlin M, Bjørn AM, Rasmussen M, Trolle B, Petersen MB (2016). Prevalence and patient characteristics of Mayer-Rokitansky-Küster-Hauser syndrome: a nationwide registry-based study. Hum Reprod.

[9] Herlin MK, Petersen MB, Brännström M (2020). Mayer-Rokitansky-Küster-Hauser (MRKH) syndrome: a comprehensive update. Orphanet J Rare Dis.

[10] Kumar S, Sharma S (2016). MURCS (Müllerian duct aplasia-renal agenesis-cervicothoracic somite dysplasia): a rare cause of primary amenorrhoea. Oxf Med Case Reports.

[11] Herlin M, Højland AT, Petersen MB (2014). Familial occurrence of Mayer-Rokitansky-Küster-Hauser syndrome: a case report and review of the literature. Am J Med Genet A.

[12] Chen N, Zhao S, Jolly A (2021). Perturbations of genes essential for Müllerian duct and Wölffian duct development in Mayer-Rokitansky-Küster-Hauser syndrome. Am J Hum Genet.

[13] (2018). ACOG Committee Opinion No. 728: Müllerian Agenesis: Diagnosis, Management, And Treatment. Obstet Gynecol.

[14] Preibsch H, Rall K, Wietek BM, Brucker SY, Staebler A, Claussen CD, Siegmann-Luz KC (2014). Clinical value of magnetic resonance imaging in patients with Mayer-Rokitansky-Küster-Hauser (MRKH) syndrome: diagnosis of associated malformations, uterine rudiments and intrauterine endometrium. Eur Radiol.

[15] Lermann J, Mueller A, Wiesinger E (2011). Comparison of different diagnostic procedures for the staging of malformations associated with Mayer-Rokitansky-Küster-Hauser syndrome. Fertil Steril.

[16] Chen N, Song S, Bao X, Zhu L (2022). Update on Mayer-Rokitansky-Küster-Hauser syndrome. Front Med.

[17] Callens N, De Cuypere G, De Sutter P, Monstrey S, Weyers S, Hoebeke P, Cools M (2014). An update on surgical and non-surgical treatments for vaginal hypoplasia. Hum Reprod Update.

